# RB1 Is an Immune-Related Prognostic Biomarker for Ovarian Cancer

**DOI:** 10.3389/fonc.2022.830908

**Published:** 2022-03-01

**Authors:** Biao Xie, Guangqing Tan, Jingyi Ren, Weiyu Lu, Sadaf Pervaz, Xinyi Ren, Antonia Adwoa Otoo, Jing Tang, Fangfang Li, Yingxiong Wang, Meijiao Wang

**Affiliations:** ^1^ Department of Biostatistics, School of Public Health and Management, Chongqing Medical University, Chongqing, China; ^2^ Department of Physiology, School of Basic Medical Science, Chongqing Medical University, Chongqing, China; ^3^ Joint International Research Laboratory of Reproduction and Development of the Ministry of Education of China, School of Public Health and Management, Chongqing Medical University, Chongqing, China; ^4^ Department of Bioinformatics, School of Basic Medical Science, Chongqing Medical University, Chongqing, China

**Keywords:** ovarian cancer, RB1, prognosis, overall survival, immune infiltration

## Abstract

**Background:**

Ovarian cancer (OC) is one of the most lethal gynecologic malignancies and a leading cause of death in the world. Thus, this necessitates identification of prognostic biomarkers which will be helpful in its treatment.

**Methods:**

The gene expression profiles from The Cancer Genome Atlas (TCGA) and GSE31245 were selected as the training cohort and validation cohort, respectively. The Kaplan–Meier (KM) survival analysis was used to analyze the difference in overall survival (OS) between high and low RB transcriptional corepressor 1 (RB1) expression groups. To confirm whether RB1 was an independent risk factor for OC, we constructed a multivariate Cox regression model. Gene Ontology (GO) terms and Kyoto Encyclopedia of Genes and Genomes (KEGG) pathways enrichment analyses were conducted to identify the functions of differentially expressed genes (DEGs). The associations of RB1 with immune infiltration and immune checkpoints were studied by the Tumor Immune Estimation Resource (TIMER 2.0) and the Gene Expression Profiling Interactive Analysis (GEPIA). The immunohistochemistry (IHC) was performed to compare the expression level of RB1 in normal tissues and tumor samples, and to predict the prognosis of OC.

**Results:**

The KM survival curve of the TCGA indicated that the OS in the high-risk group was lower than that in the low-risk group (HR = 1.61, 95% CI: 1.28-2.02, *P* = 3×10^-5^), which was validated in GSE31245 (HR = 4.08, 95% CI: 1.21–13.74, *P* = 0.01) and IHC. Multivariate Cox regression analysis revealed that RB1 was an independent prognostic biomarker (HR = 1.66, 95% CI: 1.31-2.10, *P* = 2.02×10^-5^). Enrichment analysis suggested that the DEGs were mainly involved in cell cycle, DNA replication, and mitochondrial transition. The infiltration levels of fibroblast, neutrophil, monocyte and macrophage were positively correlated with RB1. Furthermore, RB1 was associated with immune checkpoint molecules (CTLA4, LAG3, and CD274). The IHC staining revealed higher expression of RB1 in tumor tissues as compared to that in normal tissues (*P* = 0.019). Overexpression of RB1 was associated with poor prognosis of OC (*P* = 0.01).

**Conclusion:**

These findings suggest that RB1 was a novel and immune-related prognostic biomarker for OC, which may be a promising target for OC treatment.

## Introduction

Ovarian cancer (OC) is a gynecological malignancy, which is reported to be the fifth leading cause of female cancer death in the United States and ninth in China, totally ranking eighth across the world (1–3). According to the global cancer statistics, in 2020, there were 313,959 new cases (3.4% of all cancer cases) and 207,252 deaths (4.7% of all cancer deaths) (3). Despite therapeutic advancement over the last decades, the overall 5-year survival rate is only 47% (4, 5). Due to the lack of credible prognostic biomarkers, hidden symptoms, and screening strategies, approximately 70% of patients are diagnosed at an advanced stage (6, 7). Risk factors for OC include endometriosis, advancing age, a family history of OC or breast cancer, and germline mutations (8–10). Malignant epithelial ovarian carcinoma occupies 90% of cases in OC, namely, five histological subtypes: high-grade serous carcinoma (70%), endometrioid carcinoma (10%), clear-cell carcinoma (10%), mucinous carcinoma (3%) and low-grade serous (<5%) (11). There is increasing evidence which indicates that OC is an immunogenic tumor with spontaneous antitumor immune responses (12–14). Immune-related prognostic biomarkers have been reported in various studies, while some biomarkers lack experimental validation (15–17). Therefore, it is of great importance to identify a reliable and immune-related tumor marker by experimental validation.

RB transcriptional corepressor 1 (RB1) is the first tumor suppressor gene mutated in retinoblastoma (18, 19). It is a multi-functional protein that promotes cancer immunity and regulates crucial cellular activities, namely, cell cycle progression, DNA damage response, checkpoint activation and differentiation (20). A previous research has shown that RB1-mediated release of IL-6 can promote aging and recruit NKT cells in the radiation response, which is helpful for cancer immune monitoring (21). Emerging data suggests that RB1 plays an important role in different aspects of immune function, namely, determining the fate of immune progenitor and regulating innate immune response (22). Samples with co-alterations of RB1 and TP53 are enriched in immune effects and show higher T cell inflammation signature scores (23). Homologous recombination DNA repair pathway disruption and RB1 deficiency have been reported to improve the prognosis of OC patients (24, 25). Moreover, RB1 is a member of retinoblastoma proteins, namely, RBL1/p107 and RBL2/p130. The latter was found altered in various cancers, serving as a potential prognostic biomarker (26).

In this study, two cohorts and their corresponding clinical information were included. The Kaplan–Meier (KM) survival curves were combined with Cox regression analysis to study the prognostic potential of RB1 for OC. The enrichment analysis was performed to explore the biological functions of differentially expressed genes (DEGs). The gene–gene interaction network was conducted to further study RB1 involved functions. The aim of this study was to analyze the prognostic value of RB1 on OC, and to explore the correlations of RB1 with immune infiltrating cells and immune checkpoint molecules. The different patterns of RB1 in adjacent non-tumor ovarian tissues and OC samples were also analyzed.

## Materials and Methods

### Samples and Datasets

The RNA-sequencing (RNA-seq) data, including, clinical features was obtained from the UCSC website (https://xenabrowser.net/), which was used as the training set. The microarray dataset GSE31245 based on the platform GPL8300 was downloaded from the Gene Expression Omnibus (GEO) (https://www.ncbi.nlm.nih.gov/geo/), which was selected as the validation cohort. Samples lacking survival information and OS less than one day were excluded.

### Evaluation of RB1 in Predicting the Survival for OC

Patients in The Cancer Genome Atlas (TCGA) were divided into high-risk and low-risk groups based on the optional cut-off value of RB1. The KM survival curve was conducted to study whether there was a difference in OS between the two groups. We further stratified samples of TCGA by age (age ≤60 and >60), stage (stages I–II and III–IV) and grade (grades 1–2 and 3–4). The time-dependent receiver operating characteristic (ROC) curve was then used to estimate the predictive capacity of RB1 for OS. Furthermore, the predictive ability of RB1 expression level among clinical subgroups (age ≤60, advanced stage and advanced grade) was analyzed. To confirm whether RB1 was an independent risk factor RB1 for OC, univariate and multivariate Cox regression analyses were conducted on three clinical variables (age, stage and grade) of the TCGA.

The external cohort GSE31245 for survival analysis was used to verify the prognostic value of RB1. Patients were classified into high and low expression groups according to the optional cut-off value of RB1. The time-dependent ROC curve was plotted to confirm the accuracy of RB1 in predicting OS.

### Enrichment Analysis and Gene–Gene Interaction Network Construction

Student’s *t*-test and false discovery rate (FDR) were used for the identification of the DEGs related to RB1 expression. FDR <0.05 was considered as significant. GO terms and KEGG pathways enrichment analyses were then conducted to analyze the biological functions of DEGs. Based on the GeneMANIA (http://genemania.org/) (27), the RB1 interaction network was performed to further explore RB1 involved functions.

### Associations With Immune Infiltration and Immune Checkpoints

Tumor Immune Estimation Resource (TIMER 2.0) (http://timer.cistrome.org/) is a web server that is used to explore the relationship between gene expression and immune infiltration across 32 cancer types (28). Herein, we studied the association of RB1 mRNA expression with 22 immune cells. Purity-corrected Spearman’s rho values and statistical significance were shown in the scatterplots. The Gene Expression Profiling Interactive Analysis (GEPIA) (http://gepia.cancer-pku.cn/) is a comprehensive online database, which adopts a standard processing approach to study RNA-seq data of 9,736 tumors and 8,587 normal tissues based on the TCGA and the Genotype-Tissue Expression (GTEx) cohorts (29). In this study, the correlation between RB1 and immune checkpoint molecules, such as CTLA4, GZMB, LAG3, PDCD1 and CD274 (PD-L1) were analyzed with GEPIA.

### The Landscape of RB1 Expression in Normal and Tumor Tissues

The Human Protein Atlas (HPA) (https://www.proteinatlas.org/) is an interactive open access database that provides a visual platform for systematic analysis of protein expression in human organs, cells and tissues. We used this database to show the distribution of RB1 mRNA and protein expression profiles in normal and tumor tissues (30, 31).

### Tissue Microarray (TMA) and Immunohistochemistry (IHC)

TMA sections (4 µm thick), including, 45 pairs of OC samples and adjacent non-tumor ovarian tissues (9 of 90 were invalid) were purchased from Superbiotek Pharmaceutical Technology (Shanghai, China). After overnight baking at 60°C, the slides were incubated with 10 mmol/L sodium citrate buffer (pH = 6.0) in the microwave for 15 min and washed with phosphate buffered saline. Blocking of endogenous peroxidases and nonspecific antigens were done with hydrogen peroxide (3%) and normal goat serum (5%), respectively. The sections were incubated with RB1 polyclonal antibody (1:400, Proteintech, China) overnight at 4°C. Slides were then incubated with the secondary antibody (Zsbio, China) for 30 min at room temperature. Sections were reacted with diaminobenzidine for 30 s and counterstained with hematoxylin for 2 min. The results of IHC staining were independently determined by two experienced pathologists who were blind to the clinical information. The staining index of each patient was calculated according to the following formula: staining score = % of positive cells × staining intensity. Specifically, staining intensity: 0 (colorless), 1 (light-yellow), 2 (brownish-yellow), and 3 (dark-brown). Tumor cells proportion: 0 (no positive tumor cells), 1 (<10%), 2 (10–25%), 3 (26–49%), and 4 (≥50%). The final score was defined as: negative (0), weakly positive (1–4), positive (5–8), and strongly positive (9–12). The optional cutoff value (staining index = 4) was determined using ROC curve analysis. IHC-score >4 represented RB1 high expression group and IHC-score ≤4 represented RB1 low expression group.

### Statistical Analysis

All the statistical analysis was conducted in R software (version 4.0.4). Wilcoxon test was used to analyze the protein expression difference between OC specimens and adjacent normal ovarian tissues. The KM survival analysis, univariate and multivariate Cox regression analyses were conducted with the “survival” R package (version 3.2.7) and the “survminer” R package (version 0.4.9). The time-dependent ROC curves were performed with the R package “timeROC” (version 0.4). Student’s *t-*test and FDR were applied for the identification of the DEGs. The “clusterProfiler” R package (version 3.18.1) was employed to perform the functional enrichment analysis. The “GOplot” R package (version 1.0.2) and the “ggplot2” R package (version 3.3.5) were used for the enrichment analysis and comparison between adjacent normal ovarian tissues and ovarian cancer samples. The “ggpubr” R package (0.4.0) was used to compare the two subtypes of OC. A two-sided *P*-value <0.05 was considered statistically significant.

## Results

### Study Design

**Figure 1:**
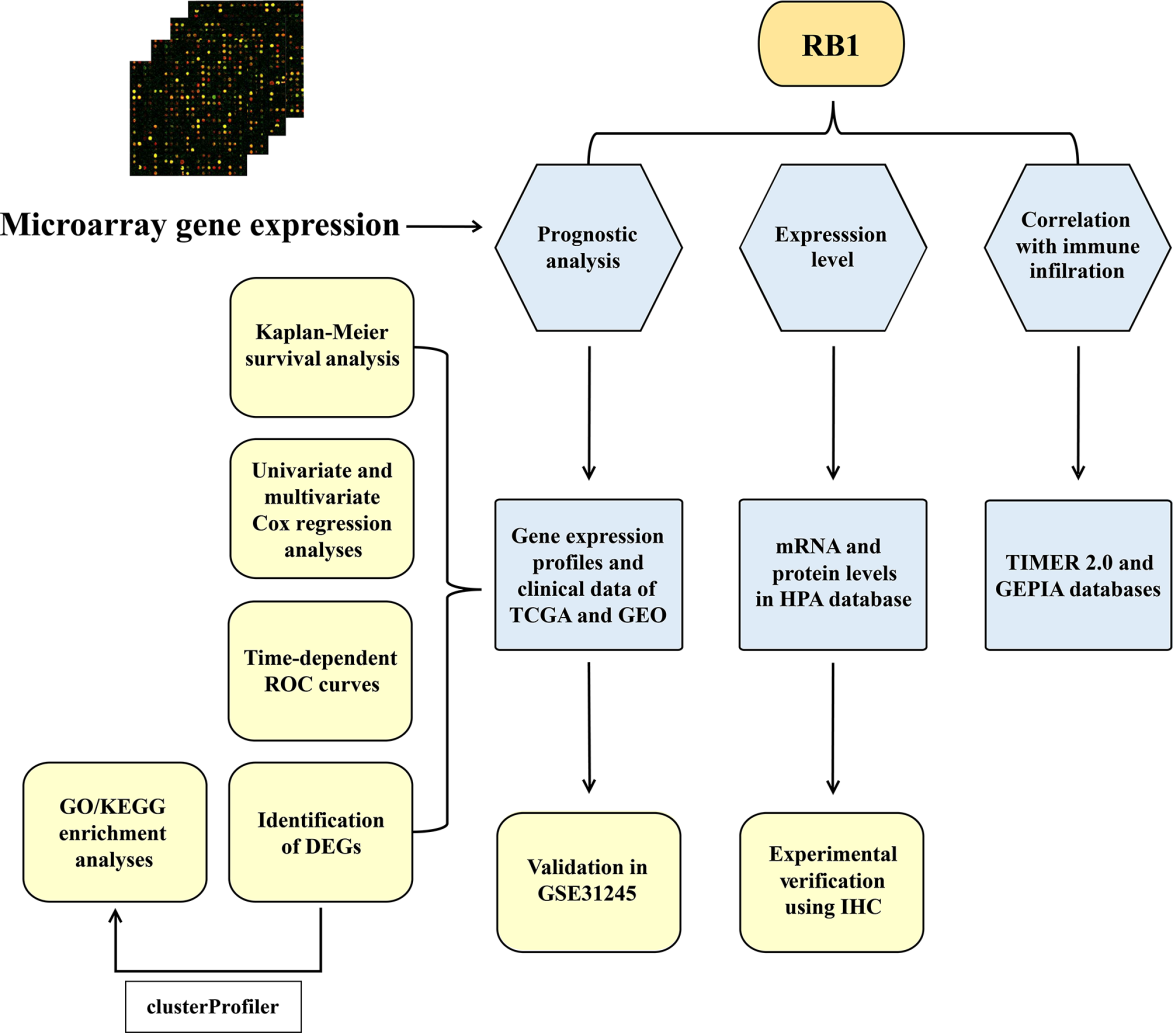
The workflow diagram of the study.

A total of 534 samples from the TCGA (training cohort) and 58 samples from GSE31245 (validation cohort) were used in this study. The workflow diagram of this study is shown in **Figure 1**.

### High Expression of RB1 Indicated Poor Prognosis for OC

**Figure 2:**
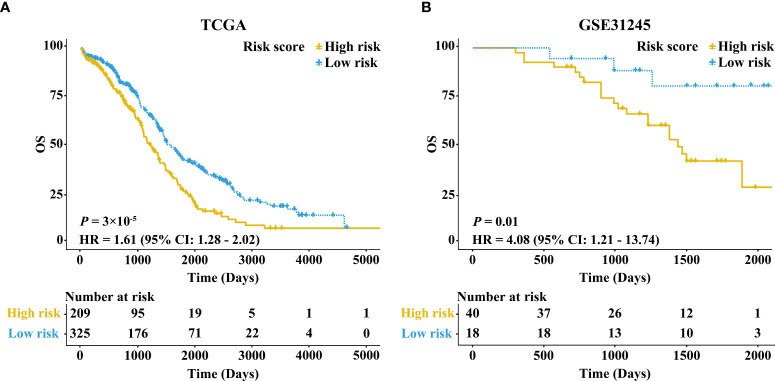
Kaplan–Meier survival curves comparing the high and low expressions of RB1 in the TCGA and GEO. **(A)** Patients with 5-year OS in the TCGA. **(B)** Patients with 5-year OS in GSE31245.

The KM survival curve showed that the OS rate of samples in the high-risk group was lower in comparison with the low-risk group (HR = 1.61, 95% CI: 1.28-2.02, *P* = 3×10^-5^) (**Figure 2A**). Taking age, stage, and grade into account, we further performed stratification analysis. Interestingly, as shown in **Supplementary Figures 1A–D**, increased RB1 was related to poor prognosis in patients at age ≤60 (HR = 1.47, 95% CI: 1.07-2.01, *P* = 0.02), advanced stage (HR = 1.66, 95% CI: 1.32-2.10, *P* = 1×10^-5^) and advanced grade (HR = 1.66, 95% CI: 1.30-2.13, *P* = 5×10^-5^). The time-dependent ROC curve suggested that RB1 (1-year AUC = 0.567, 3-year AUC = 0.574, 5-year AUC = 0.596) had a certain capacity to predict the prognosis of OC in the TCGA (**Supplementary Figure 2A**). The results revealed that patients at age ≤60 (1-year AUC = 0.513, 3-year AUC = 0.544, 5-year AUC = 0.556), advanced stage (1-year AUC = 0.585, 3-year AUC = 0.579, 5-year AUC = 0.597) and advanced grade (1-year AUC = 0.567, 3-year AUC = 0.582, 5-year AUC = 0.615) were associated with poor prognosis (**Supplementary Figures 2B–D**).

**Figure 3:**
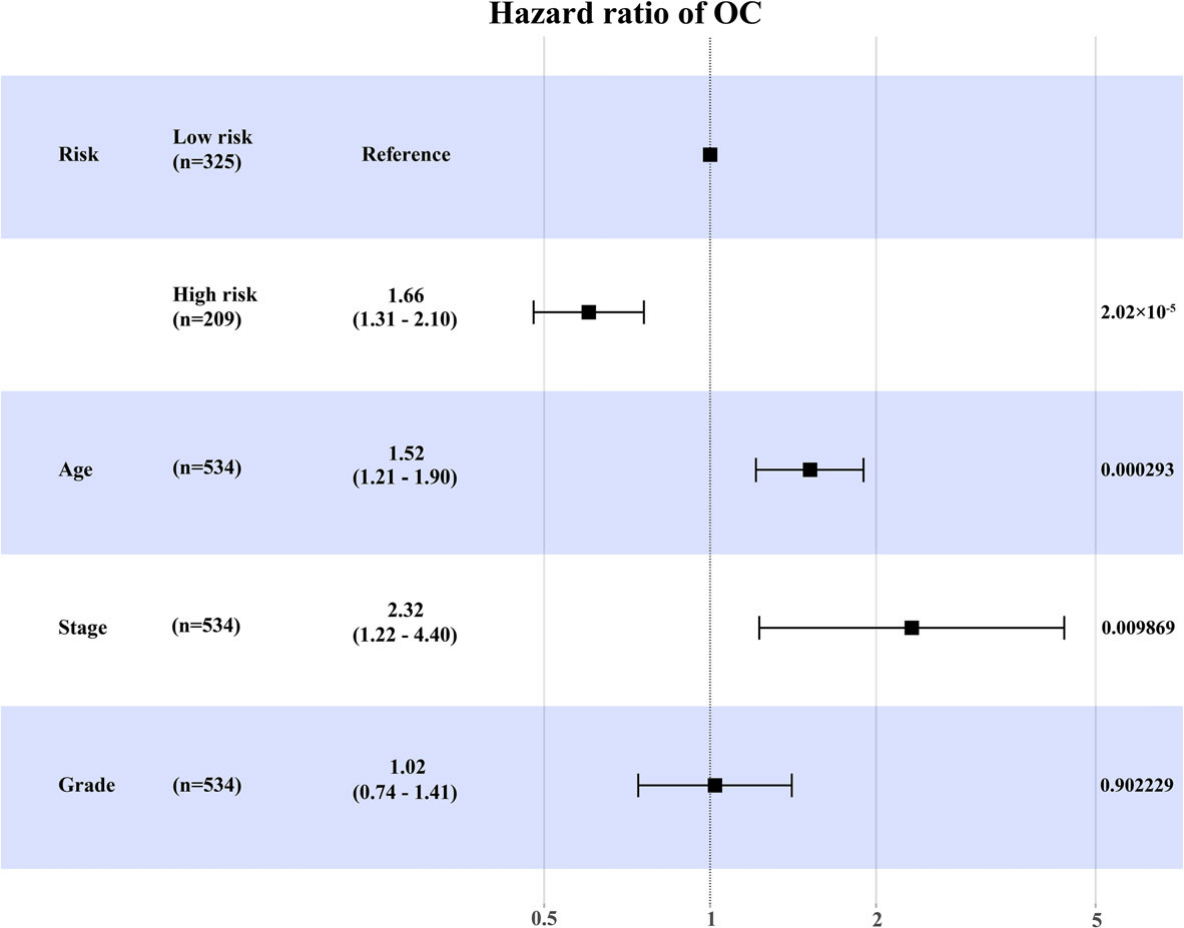
The forest map to visualize the multivariate Cox regression analysis incorporating RB1 into clinical variables in the TCGA.

Besides, we further conducted univariate and multivariate Cox regression analyses in the training set (**Supplementary Table 1**). Multivariate Cox regression analysis showed that the expression level of RB1 (HR = 1.66, 95% CI: 1.31-2.10, *P* = 2.02×10^-5^) and age (HR = 1.52, 95% CI:1.21-1.90, *P* = 0.000293) were independent prognostic factors for OC (**Figure 3**).

The results in external validation dataset GSE31245 revealed that increased RB1 indicated poor prognosis (HR = 4.08, 95% CI: 1.21–13.74, *P* = 0.01), which was shown in **Figure 2B**. The time-dependent ROC curve proved that RB1 was effective and robust in predicting the prognosis of OC, the AUCs at 1-, 3- and 5-years were 0.752, 0.664, and 0.715 respectively (**Supplementary Figure 2E**).

### Functional Enrichment Analysis and RB1-Interacting Genes

**Figure 4:**
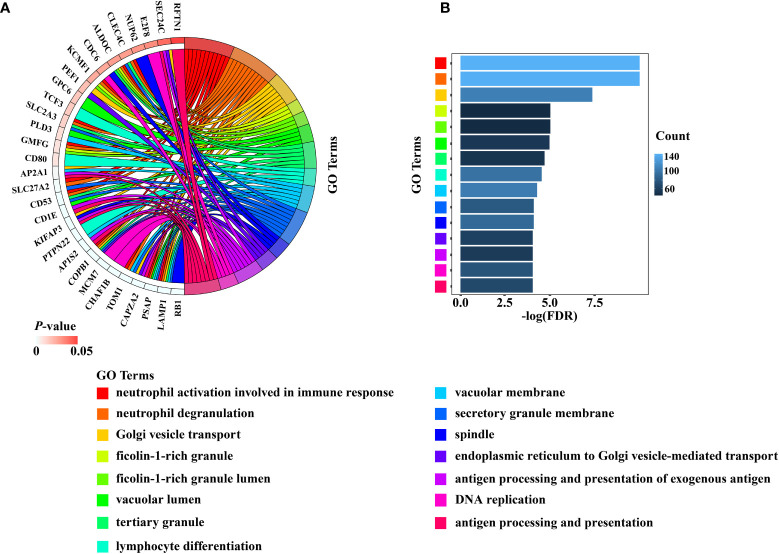
The results of the top 15 significant GO terms of BP and CC were presented by **(A)** Chord diagram, **(B)** Histogram.

The total number of enriched terms was 455 (**Supplementary Table 2**). Arranged in ascending order of FDR, the top 15 significant GO terms of biological process (BP) and cellular component (CC) indicated that the DEGs mainly participated in cell cycle, DNA replication, mitochondrial transition and immune response (**Figures 4A, B** **)**. The GeneMANIA results included 20 RB1-interacting genes, among which CCND1, MYC, CBX4, KDM6A, PPP2R3B, RBL1, TAF1, RBL2, RBP2, and ATF7 were tightly interconnected around (**Supplementary Figure 3**).

### Correlations With Immune Infiltration and Immune Checkpoints

**Figure 5:**
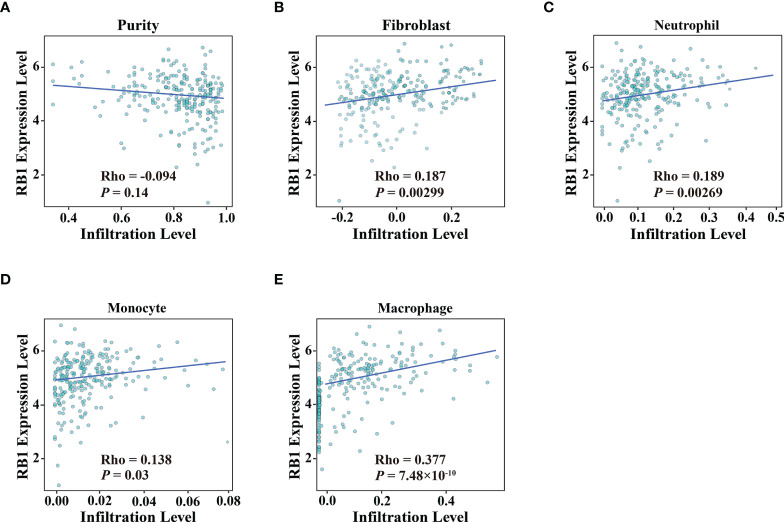
Association of RB1 expression and immune infiltration in OC was available at TIMER 2.0. **(A)** Tumor purity, **(B)** Fibroblast, **(C)** Neutrophil, **(D)** Monocyte, **(E)** Macrophage.

**Figure 6:**
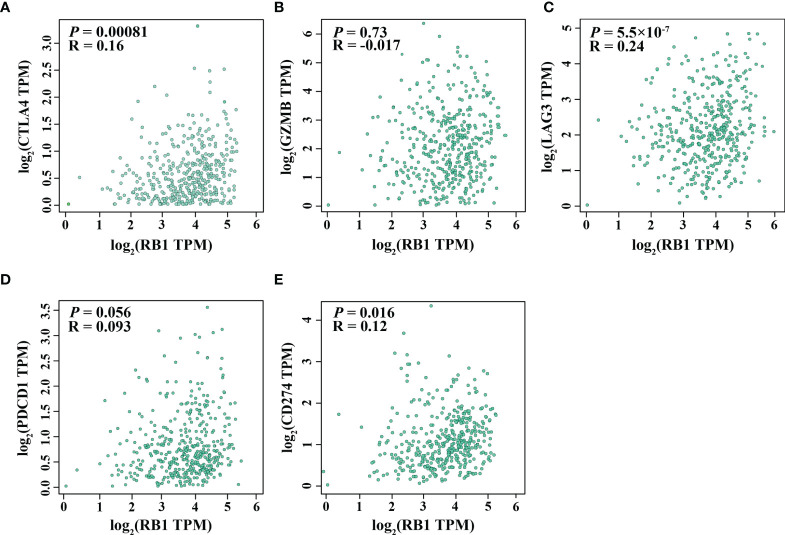
Correlation between RB1 expression and T cell exhaustion in OC (GEPIA). **(A)** CTLA4, **(B)** GZMB, **(C)** LAG3, **(D)** PDCD1, **(E)** CD274.

Using the online website TIMER 2.0, we discovered that the infiltration levels of fibroblast, neutrophil, monocyte and macrophage were positively correlated with RB1 expression (*P <*0.05, **Figures 5A–E**). Results from GEPIA indicated a positive association of RB1 expression with CTLA4, LAG3 and CD274 in tumor tissues (*P <*0.05, **Figures 6A, C, E** **)**, whereas there was no significant association with other immune checkpoint molecules (**Figures 6B, D** **)**.

### The Expression Level of RB1 in Normal and Tumor Tissues

The characteristics of RB1 expression were shown in HPA. RB1 represented a relatively lower expression level in ovarian tissues compared with other normal organs and tumor tissues, as shown in **Supplementary Figures 4A, B**. However, the protein expression of RB1 in ovarian tissues was higher than that in other normal organs and tumor tissues (**Supplementary Figures 4C, D** **)**.

### Experimental Validation

**Figure 7:**
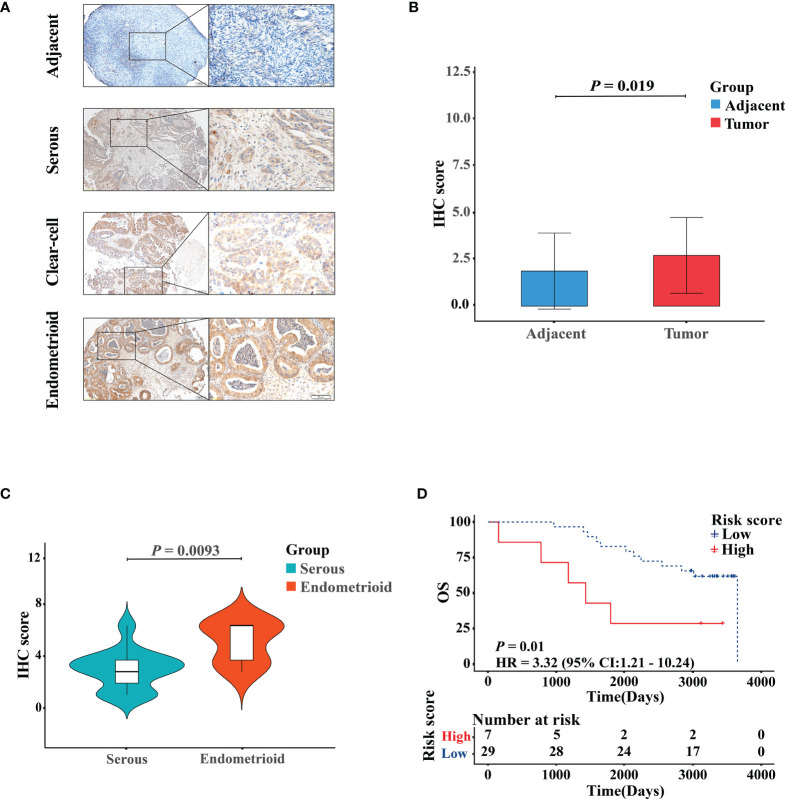
Validation of the protein expression and prognostic performance of RB1 in patients with OC by IHC. **(A)** IHC detection of RB1 expression in adjacent normal ovarian tissues, serous, clear-cell, and endometrioid ovarian cancers. Representative images were shown at ×10 (scale bar, 200 μm) and ×40 (scale bar, 50 μm) *via* microscope. **(B)** Staining index of RB1 between adjacent normal tissues and tumor tissues. **(C)** RB1 expression in different histologic subtypes of OC was detected by IHC analysis. **(D)** KM survival analysis was performed on 36 TMA tumor specimens to study the difference of OS between RB1 high and low expression groups.

The representative images of the immunohistochemical staining of RB1 on the TMA between OC specimens and adjacent non-tumor ovarian tissues were shown in **Figure 7A**. The protein expression level of RB1 was upregulated in tumor tissues than that in adjacent normal tissues and was mainly localized in the nucleus (*P* = 0.019, **Figure 7B**). The correlation between RB1 expression and histological subtypes was confirmed. The protein level of RB1 in endometrioid ovarian cancer was higher compared with serous ovarian carcinoma (*P <*0.01, **Figure 7C**). Despite there was only one case of clear-cell ovarian carcinoma sample, RB1 expression was higher than that in serous ovarian carcinoma. Correlation analyses revealed that RB1 expression had no significant relationship with age, stage and the recurrence of state (**Supplementary Table 3**). KM survival curve indicated that overexpression of RB1 was correlated with poor outcomes (**Figure 7D**), which was consistent with the KM survival curves of the TCGA and GSE31245.

## Discussion

RB1 is involved in the pathogenesis of various cancers (32, 33). Besides causing retinoblastoma (34), mutations and inactivation of RB1 occur in osteosarcoma (35), lung (36), bladder (37), esophageal (38), and breast (39) malignancies. Notably, recent studies have found that RB1 deficiency can predict drug resistance and sensitivity in triple-negative breast (40), pancreatic (41), and prostate (42) cancers. A previous study has reported that RB1 loss is correlated with poor prognosis in glioblastoma (43) and small cell lung cancer (44). However, high expression of RB1 is associated with poor prognosis in advanced-stage ovarian carcinoma patients (45, 46). HPA database has mentioned that RB1 is an unfavorable prognostic factor for OC. In this study, the result of univariate and multivariate Cox regression analyses demonstrated that RB1 was an independent prognostic factor for OC. Based on the KM survival analysis, we concluded that overexpression of RB1 indicated poor prognosis for OC, which was verified by external validation dataset and IHC. Furthermore, RB1 was positively associated with immune infiltrating cells and immune checkpoint molecules.

In this study, we found that the protein level of RB1 was higher in OC tissues than that in adjacent non-tumor ovarian samples. Milde-Langosch et al. (47) also demonstrated that expression of RB1 was below the limits of detection in normal ovarian tissues by immunohistochemical staining, in comparison with high expression level in OC patients. According to the IHC-score, the KM survival curve proved that overexpression of RB1 was correlated with a shorter OS, which was consistent with the result of analyses using the HPA database and two cohorts. Furthermore, the relationship between RB1 expression and the clinical features of samples with OC was studied. We found that a higher expression level of RB1 was observed in endometrioid ovarian cancer than that in serous ovarian cancer. Although there was only one case of clear-cell ovarian cancer, RB1 expression was higher in clear-cell ovarian cancer than that in serous ovarian cancer. Results from the stratification analysis showed a difference in the prognosis between patients with high and low expression levels of RB1 at age ≤60, advanced stage and advanced grade, which was consistent with the previous studies (48, 49). The above results suggested that RB1 played an important role in the progression and metastasis of OC, providing targeted guidance for the treatment of patients at an early age, advanced stage and advanced grade.

GO and KEGG analyses indicated that the RB1-related genes were primarily involved in cell cycle, DNA replication and mitochondrial transition. Similar results were obtained in the gene–gene interaction network, where a strong confidence correlation was found between RB1 and transport proteins. For instance, RB1 interacted with CCND1 to regulate the division cycle of G1/S transition. From the Genecards and UniProt online websites, we discovered that RB1 acted as a regulator of the entry into cell division through interacting with E2F family and repressing the transcriptional activity of responsive genes (50). As a result, the cell cycle was arrested and the cell proliferation were restrained (51). Furthermore, overexpression of RB1 interfered with the regulation of cell cycle, playing a critical role in the occurrence and development of cancer (52, 53).

Tumor-infiltrating lymphocytes in the immune microenvironment have been proven to be a prognostic factor for various cancers (54). A high M1/M2 ratio of tumor-associated macrophages can prolong the survival time of OC (55, 56). Our study revealed that RB1 had a positive correlation with immune infiltrating cells and immune checkpoint molecules. Data has shown that RB1 exists in S249/T252 in the form of CDK4/6 phosphorylation, which can counteract cancer immune evasion (20). Specifically, RB1 can downregulate the transcriptional target of nuclear factor-kappa B (NF-κB) *via* interacting with NF-κB to promote tumor immunity. These downregulated NF-κB targets also include PD-L1, a key immune checkpoint factor, whose abnormal expression in tumor cells suppresses cancer immunity through the binding of PD-L1 to its homologous receptor PD-1 on T cells, resulting in its inactivation and apoptosis (20, 33). Taken together, these findings suggest that RB1 may play a role in regulating the immune microenvironment of OC.

To note, there are limitations in this study. Firstly, although functional enrichment analysis and gene–gene interaction network were performed, further functional experiments of RB1 on OC progression will be required. Secondly, the sample size of this study may be insufficient, so an enlargement of the sample size is required to verify the results.

## Conclusion

In conclusion, our study revealed that RB1 was a robust and immune-related prognostic biomarker for OC. The enrichment pathways of RB1-related genes and its correlation with immune infiltration in the immune microenvironment suggested that RB1 may be a promising novel therapeutic target for OC immunotherapy.

## Data Availability Statement

The datasets presented in this study can be found in online repositories. The names of the repository/repositories and accession number(s) can be found in the article/**Supplementary Material**.

## Ethics Statement

The studies involving human participants were reviewed and approved by The Ethics Committee of the Chongqing Medical University. The patients/participants provided their written informed consent to participate in this study. Written informed consent was obtained from the individual(s) for the publication of any potentially identifiable images or data included in this article.

## Author Contributions

BX and GT conceived and designed this study. BX collected and analyzed the relative data. GT wrote the manuscript. SP and MW conducted the IHC experiments. MW, BX, JR, WL, XR, AO, JT, FL and YW revised the manuscript. MW supervised the whole study. All authors listed have made a substantial, direct, and intellectual contribution to the work and approved it for publication.

## Funding

This study was supported by grants from the National Natural Science Foundation of China (No. 82171624), the Chongqing Natural Science Foundation (No. cstc2020jcyj-msxmX0294), the Science and Technology Project of Chongqing Yuzhong District (No. 20200103), and the Scientific Research & Innovation Experiment Project of Chongqing Medical University (SRIEP202002, SRIEP202106).

## Conflict of Interest

The authors declare that the research was conducted in the absence of any commercial or financial relationships that could be construed as a potential conflict of interest.

## Publisher’s Note

All claims expressed in this article are solely those of the authors and do not necessarily represent those of their affiliated organizations, or those of the publisher, the editors and the reviewers. Any product that may be evaluated in this article, or claim that may be made by its manufacturer, is not guaranteed or endorsed by the publisher.

## Supplementary Material

Supplementary Figure 1 Stratification analysis. KM analysis of OC patients in TCGA stratified by risk scores combined with **(A)** Age ≤ 60. **(B)** Age > 60. **(C)** Early stage. **(D)** Advanced stage. **(E)** Early grade. **(F)** Advanced grade.

Supplementary Figure 2 Verification of the accuracy of RB1 in predicating OS and its expression level among different clinical subgroups was analyzed. **(A)** The AUCs at 1, 3 and 5 years in TCGA. **(B)** Age ≤ 60, **(C)** Advanced stage and **(D)** Advanced grade. **(E)** The AUCs at 1, 3 and 5 years in GSE31245.

Supplementary Figure 3 Gene-Gene interaction network, which was related to RB1.

Supplementary Figure 4 RB1 expression profiles in human normal and cancer tissues. **(A)** mRNA expression data from the GTEx project. **(B)** Gene expression in common human tumor tissues. **(C)** Protein expression of normal tissues in different organs. **(D)** Protein expression overview in common tumors.
