# Supplementary figures and images for: RB1 Is an Immune-Related Prognostic Biomarker for Ovarian Cancer
Source: Front Oncol. 2022 Mar 1;12:830908. doi: 10.3389/fonc.2022.830908 (PMC8920998; doi:10.3389/fonc.2022.830908)

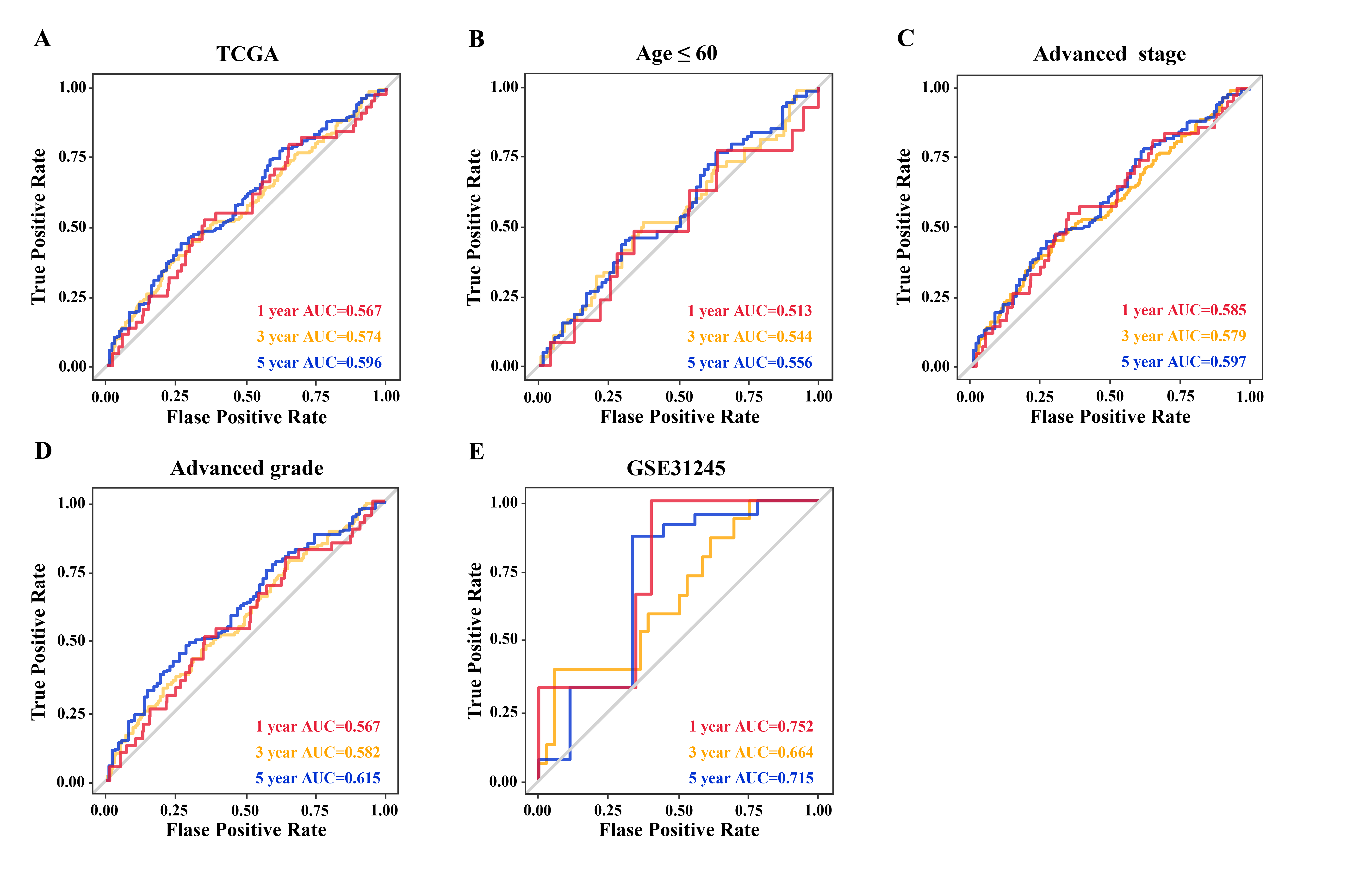

Supplement: Supplementary Figure 2 — Verification of the accuracy of RB1 in predicating OS and its expression level among different clinical subgroups was analyzed. (A) The AUCs at 1, 3 and 5 years in TCGA. (B) Age ≤ 60, (C) Advanced stage and (D) Advanced grade. (E) The AUCs at 1, 3 and 5 years in GSE31245. [file Image_2.tif]

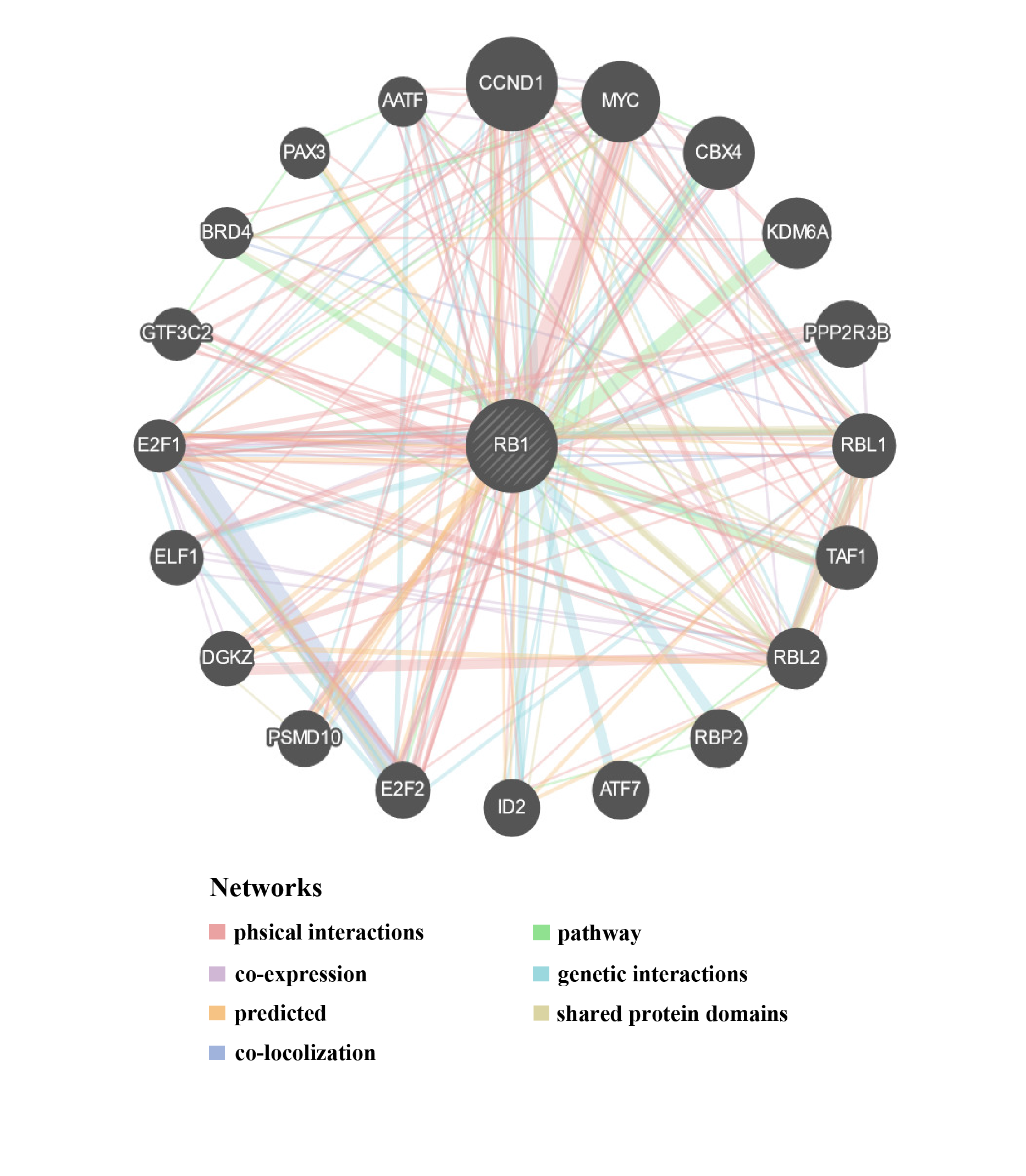

Supplement: Supplementary Figure 3 — Gene-Gene interaction network, which was related to RB1. [file Image_3.tif]

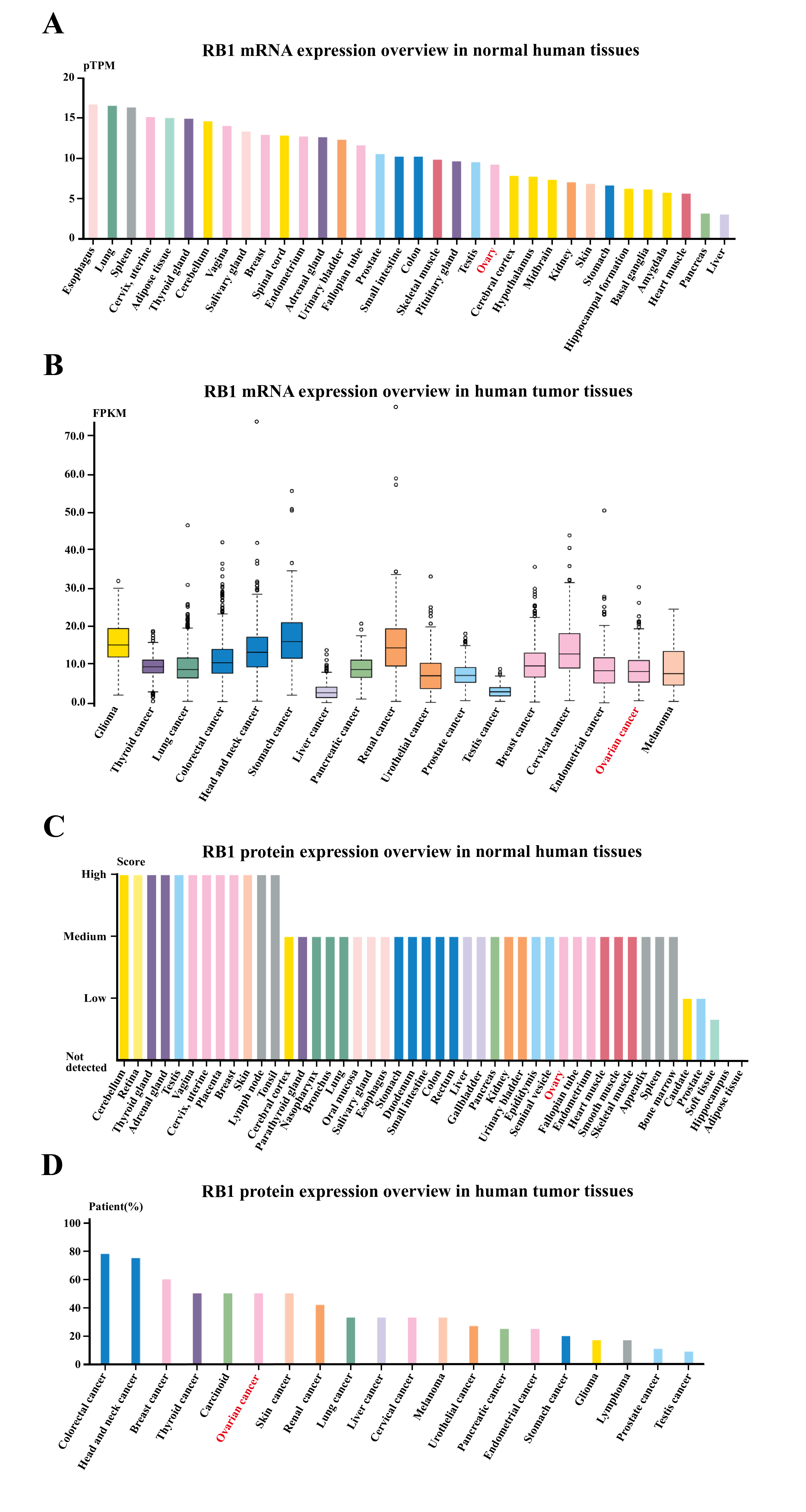

Supplement: Supplementary Figure 4 — RB1 expression profiles in human normal and cancer tissues. (A) mRNA expression data from the GTEx project. (B) Gene expression in common human tumor tissues. (C) Protein expression of normal tissues in different organs. (D) Protein expression overview in common tumors. [file Image_4.tif]
