# Supplementary material for: RB1 Is an Immune-Related Prognostic Biomarker for Ovarian Cancer
Source: Front Oncol. 2022 Mar 1;12:830908. doi: 10.3389/fonc.2022.830908 (PMC8920998; doi:10.3389/fonc.2022.830908)
Supplement: Supplementary file 5 [file Table_1.docx]

**Supplementary Table 1. Univariate and multivariate analyses of prognostic factors in terms of OS.**

| **Variable** | **Univariate analysis** | |  | | | **Multivariate analysis** | |
| --- | --- | --- | --- | --- | --- | --- | --- |
|  | **HR (95% CI)** | ***P*-value** | | |  | **HR (95% CI)** | ***P*-value** |
| **Age** | 1.48 (1.18 - 1.84) | 0.000614 | |  | | 1.52 (1.21 - 1.90) | 0.000293 |
| **Stage** | 2.42 (1.29 - 4.54) | 0.00615 | |  | | 2.32 (1.22 - 4.40) | 0.009869 |
| **Grade** | 1.19 (0.86 - 1.63) | 0.29400 | |  | | 1.02 (0.74 - 1.41) | 0.902229 |
| **Risk** | 1.61 (1.28 - 2.02) | 3.78×10^-5^ | |  | | 1.66 (1.31 - 2.10) | 2.02×10^-5^ |

OS, overall survival. HR, hazard ratio; CI, confidence interval.
