# Supplementary material for: RB1 Is an Immune-Related Prognostic Biomarker for Ovarian Cancer
Source: Front Oncol. 2022 Mar 1;12:830908. doi: 10.3389/fonc.2022.830908 (PMC8920998; doi:10.3389/fonc.2022.830908)
Supplement: Supplementary file 6 [file Table_2.docx]

**Supplementary Table 2. GO and KEGG enrichment results of the DEGs.**

| **ID** | **Description** | ***P*-value** | **FDR** | **Count** |
| --- | --- | --- | --- | --- |
| GO:0002283 | neutrophil activation involved in immune response | 2.48E-14 | 1.06E-10 | 141 |
| GO:0043312 | neutrophil degranulation | 3.34E-14 | 1.06E-10 | 140 |
| GO:0048193 | Golgi vesicle transport | 2.17E-11 | 4.58E-08 | 108 |
| GO:0101002 | ficolin-1-rich granule | 2.73E-08 | 1.00E-05 | 44 |
| GO:1904813 | ficolin-1-rich granule lumen | 2.73E-08 | 1.00E-05 | 44 |
| GO:0005775 | vacuolar lumen | 4.50E-08 | 1.11E-05 | 55 |
| GO:0070820 | tertiary granule | 1.14E-07 | 2.10E-05 | 52 |
| GO:0030098 | lymphocyte differentiation | 1.91E-08 | 3.02E-05 | 98 |
| GO:0005774 | vacuolar membrane | 3.72E-07 | 5.48E-05 | 105 |
| GO:0030667 | secretory granule membrane | 6.92E-07 | 8.34E-05 | 80 |
| GO:0005819 | spindle | 7.93E-07 | 8.34E-05 | 92 |
| GO:0006888 | endoplasmic reticulum to Golgi vesicle-mediated transport | 7.58E-08 | 9.49E-05 | 62 |
| GO:0019884 | antigen processing and presentation of exogenous antigen | 1.04E-07 | 9.49E-05 | 57 |
| GO:0006260 | DNA replication | 1.06E-07 | 9.49E-05 | 76 |
| GO:0019882 | antigen processing and presentation | 1.33E-07 | 9.49E-05 | 67 |
| GO:0042110 | T cell activation | 1.35E-07 | 9.49E-05 | 118 |
| hsa03030 | DNA replication | 3.63E-07 | 0.0001198 | 20 |
| GO:0032944 | regulation of mononuclear cell proliferation | 2.26E-07 | 0.0001366 | 64 |
| GO:0070663 | regulation of leukocyte proliferation | 2.38E-07 | 0.0001366 | 68 |
| GO:0005765 | lysosomal membrane | 2.13E-06 | 0.0001746 | 92 |
| GO:0098852 | lytic vacuole membrane | 2.13E-06 | 0.0001746 | 92 |
| GO:0035579 | specific granule membrane | 2.58E-06 | 0.0001899 | 32 |
| GO:0007249 | I-kappaB kinase/NF-kappaB signaling | 3.86E-07 | 0.0002031 | 77 |
| GO:0043122 | regulation of I-kappaB kinase/NF-kappaB signaling | 7.82E-07 | 0.000304 | 69 |
| GO:0001819 | positive regulation of cytokine production | 8.06E-07 | 0.000304 | 108 |
| GO:0050670 | regulation of lymphocyte proliferation | 8.18E-07 | 0.000304 | 62 |
| GO:0002495 | antigen processing and presentation of peptide antigen via MHC class II | 8.34E-07 | 0.000304 | 36 |
| GO:0010256 | endomembrane system organization | 8.37E-07 | 0.000304 | 112 |
| GO:0019886 | antigen processing and presentation of exogenous peptide antigen via MHC class II | 8.66E-07 | 0.000304 | 35 |
| GO:0050863 | regulation of T cell activation | 9.94E-07 | 0.0003308 | 85 |
| GO:0002504 | antigen processing and presentation of peptide or polysaccharide antigen via MHC class II | 1.09E-06 | 0.0003436 | 36 |
| GO:0044786 | cell cycle DNA replication | 1.52E-06 | 0.000458 | 26 |
| GO:0043202 | lysosomal lumen | 9.37E-06 | 0.0006278 | 32 |
| GO:0005874 | microtubule | 1.06E-05 | 0.0006495 | 100 |
| GO:0051983 | regulation of chromosome segregation | 2.34E-06 | 0.0006721 | 36 |
| GO:0030135 | coated vesicle | 1.30E-05 | 0.0007375 | 74 |
| GO:0016236 | macroautophagy | 2.86E-06 | 0.0007862 | 79 |
| GO:0033044 | regulation of chromosome organization | 3.09E-06 | 0.0007868 | 88 |
| GO:0045785 | positive regulation of cell adhesion | 3.11E-06 | 0.0007868 | 102 |
| GO:0042129 | regulation of T cell proliferation | 3.76E-06 | 0.0008608 | 49 |
| GO:0042098 | T cell proliferation | 3.77E-06 | 0.0008608 | 55 |
| GO:0033260 | nuclear DNA replication | 3.81E-06 | 0.0008608 | 24 |
| GO:0070661 | leukocyte proliferation | 4.23E-06 | 0.0009057 | 79 |
| GO:0002478 | antigen processing and presentation of exogenous peptide antigen | 4.30E-06 | 0.0009057 | 51 |
| GO:0030496 | midbody | 1.95E-05 | 0.0009579 | 50 |
| GO:0098589 | membrane region | 2.06E-05 | 0.0009579 | 82 |
| GO:0045335 | phagocytic vesicle | 2.10E-05 | 0.0009579 | 41 |
| GO:0035578 | azurophil granule lumen | 2.24E-05 | 0.0009579 | 30 |
| GO:0005667 | transcription regulator complex | 2.49E-05 | 0.0009579 | 95 |
| GO:0098687 | chromosomal region | 2.52E-05 | 0.0009579 | 83 |
| GO:0042470 | melanosome | 2.73E-05 | 0.0009579 | 33 |
| GO:0048770 | pigment granule | 2.73E-05 | 0.0009579 | 33 |
| GO:0005657 | replication fork | 3.04E-05 | 0.0010178 | 25 |
| GO:0070997 | neuron death | 5.00E-06 | 0.00102 | 88 |
| GO:1902105 | regulation of leukocyte differentiation | 5.53E-06 | 0.0010926 | 74 |
| GO:0007033 | vacuole organization | 5.78E-06 | 0.0011063 | 50 |
| GO:0032943 | mononuclear cell proliferation | 6.31E-06 | 0.00117 | 73 |
| GO:0007035 | vacuolar acidification | 6.91E-06 | 0.00117 | 13 |
| GO:0000727 | double-strand break repair via break-induced replication | 7.01E-06 | 0.00117 | 9 |
| GO:0032392 | DNA geometric change | 7.03E-06 | 0.00117 | 37 |
| GO:0048002 | antigen processing and presentation of peptide antigen | 7.04E-06 | 0.00117 | 54 |
| GO:0032386 | regulation of intracellular transport | 7.22E-06 | 0.00117 | 87 |
| GO:0045121 | membrane raft | 4.39E-05 | 0.001408 | 78 |
| GO:0098857 | membrane microdomain | 4.90E-05 | 0.0015052 | 78 |
| GO:0051452 | intracellular pH reduction | 9.58E-06 | 0.0015139 | 22 |
| GO:0051169 | nuclear transport | 1.16E-05 | 0.0017933 | 86 |
| hsa04142 | Lysosome | 1.11E-05 | 0.0018331 | 44 |
| GO:0033045 | regulation of sister chromatid segregation | 1.24E-05 | 0.0018406 | 29 |
| GO:0043123 | positive regulation of I-kappaB kinase/NF-kappaB signaling | 1.25E-05 | 0.0018406 | 52 |
| GO:0000281 | mitotic cytokinesis | 1.39E-05 | 0.0019915 | 26 |
| GO:0046651 | lymphocyte proliferation | 1.57E-05 | 0.0021312 | 71 |
| GO:0000070 | mitotic sister chromatid segregation | 1.58E-05 | 0.0021312 | 46 |
| GO:0030217 | T cell differentiation | 1.58E-05 | 0.0021312 | 65 |
| GO:0005798 | Golgi-associated vesicle | 7.44E-05 | 0.0021948 | 48 |
| GO:0050870 | positive regulation of T cell activation | 1.68E-05 | 0.0022002 | 57 |
| GO:0022407 | regulation of cell-cell adhesion | 1.71E-05 | 0.0022002 | 101 |
| GO:0045619 | regulation of lymphocyte differentiation | 1.89E-05 | 0.002391 | 50 |
| GO:0032147 | activation of protein kinase activity | 2.04E-05 | 0.0025276 | 80 |
| GO:1901214 | regulation of neuron death | 2.11E-05 | 0.0025603 | 78 |
| GO:0061695 | transferase complex, transferring phosphorus-containing groups | 9.47E-05 | 0.0026836 | 62 |
| GO:0030662 | coated vesicle membrane | 9.97E-05 | 0.0027222 | 48 |
| GO:0140014 | mitotic nuclear division | 2.29E-05 | 0.002736 | 71 |
| GO:0008287 | protein serine/threonine phosphatase complex | 0.0001123 | 0.0027728 | 19 |
| GO:1903293 | phosphatase complex | 0.0001123 | 0.0027728 | 19 |
| GO:0005766 | primary lysosome | 0.0001213 | 0.0027728 | 42 |
| GO:0042582 | azurophil granule | 0.0001213 | 0.0027728 | 42 |
| GO:0005802 | trans-Golgi network | 0.000124 | 0.0027728 | 60 |
| GO:0042581 | specific granule | 0.0001242 | 0.0027728 | 43 |
| GO:0051656 | establishment of organelle localization | 2.43E-05 | 0.0028482 | 99 |
| GO:0006268 | DNA unwinding involved in DNA replication | 2.49E-05 | 0.0028672 | 10 |
| GO:0045851 | pH reduction | 2.59E-05 | 0.0029199 | 22 |
| GO:0006913 | nucleocytoplasmic transport | 2.64E-05 | 0.0029274 | 84 |
| GO:0032606 | type I interferon production | 2.73E-05 | 0.0029792 | 38 |
| GO:0030670 | phagocytic vesicle membrane | 0.0001385 | 0.0030029 | 25 |
| GO:0070821 | tertiary granule membrane | 0.0001504 | 0.0031661 | 24 |
| GO:0032946 | positive regulation of mononuclear cell proliferation | 3.19E-05 | 0.0033737 | 40 |
| GO:0006261 | DNA-dependent DNA replication | 3.20E-05 | 0.0033737 | 43 |
| GO:0042102 | positive regulation of T cell proliferation | 3.31E-05 | 0.0034331 | 32 |
| GO:0060205 | cytoplasmic vesicle lumen | 0.0001686 | 0.0034513 | 75 |
| GO:0008347 | glial cell migration | 3.50E-05 | 0.0035658 | 21 |
| GO:0050764 | regulation of phagocytosis | 3.62E-05 | 0.0036329 | 31 |
| GO:0002683 | negative regulation of immune system process | 3.68E-05 | 0.0036372 | 104 |
| hsa04145 | Phagosome | 5.08E-05 | 0.0037766 | 47 |
| hsa05212 | Pancreatic cancer | 5.99E-05 | 0.0037766 | 28 |
| hsa04380 | Osteoclast differentiation | 6.23E-05 | 0.0037766 | 41 |
| hsa04140 | Autophagy - animal | 6.87E-05 | 0.0037766 | 44 |
| GO:0034774 | secretory granule lumen | 0.0001922 | 0.0038281 | 74 |
| GO:0031983 | vesicle lumen | 0.0002059 | 0.0039936 | 75 |
| GO:0030666 | endocytic vesicle membrane | 0.0002252 | 0.004256 | 43 |
| GO:0032479 | regulation of type I interferon production | 4.49E-05 | 0.0043101 | 37 |
| GO:0071216 | cellular response to biotic stimulus | 4.56E-05 | 0.0043101 | 62 |
| GO:0045787 | positive regulation of cell cycle | 4.57E-05 | 0.0043101 | 91 |
| GO:0061900 | glial cell activation | 4.76E-05 | 0.0044269 | 22 |
| GO:0007088 | regulation of mitotic nuclear division | 4.90E-05 | 0.0044883 | 45 |
| GO:0050866 | negative regulation of cell activation | 5.12E-05 | 0.0046194 | 54 |
| GO:1903037 | regulation of leukocyte cell-cell adhesion | 5.19E-05 | 0.004622 | 78 |
| GO:0098791 | Golgi apparatus subcompartment | 0.0002617 | 0.0047785 | 82 |
| GO:0030660 | Golgi-associated vesicle membrane | 0.0002658 | 0.0047785 | 32 |
| GO:0032508 | DNA duplex unwinding | 5.68E-05 | 0.004984 | 33 |
| GO:0070665 | positive regulation of leukocyte proliferation | 6.04E-05 | 0.0052324 | 42 |
| GO:0050671 | positive regulation of lymphocyte proliferation | 6.17E-05 | 0.0052697 | 39 |
| GO:0002695 | negative regulation of leukocyte activation | 6.37E-05 | 0.0053712 | 49 |
| GO:0033176 | proton-transporting V-type ATPase complex | 0.0003085 | 0.0054127 | 12 |
| GO:1902115 | regulation of organelle assembly | 6.58E-05 | 0.0054698 | 51 |
| GO:0031984 | organelle subcompartment | 0.0003225 | 0.0055267 | 86 |
| GO:0000075 | cell cycle checkpoint | 6.81E-05 | 0.0055917 | 56 |
| GO:1904724 | tertiary granule lumen | 0.0003509 | 0.0057564 | 19 |
| GO:0005925 | focal adhesion | 0.0003567 | 0.0057564 | 90 |
| GO:0016363 | nuclear matrix | 0.0003593 | 0.0057564 | 31 |
| hsa04071 | Sphingolipid signaling pathway | 0.000123 | 0.0057968 | 38 |
| GO:0007173 | epidermal growth factor receptor signaling pathway | 7.32E-05 | 0.0059322 | 36 |
| GO:0007159 | leukocyte cell-cell adhesion | 7.53E-05 | 0.0060073 | 84 |
| GO:0051402 | neuron apoptotic process | 7.63E-05 | 0.0060073 | 61 |
| GO:1903039 | positive regulation of leukocyte cell-cell adhesion | 7.73E-05 | 0.0060073 | 59 |
| GO:0072659 | protein localization to plasma membrane | 7.89E-05 | 0.0060073 | 68 |
| GO:0007093 | mitotic cell cycle checkpoint | 7.89E-05 | 0.0060073 | 45 |
| GO:1905818 | regulation of chromosome separation | 8.11E-05 | 0.0060996 | 23 |
| GO:0001772 | immunological synapse | 0.0003893 | 0.006104 | 15 |
| GO:0001774 | microglial cell activation | 8.80E-05 | 0.0064716 | 19 |
| GO:0002269 | leukocyte activation involved in inflammatory response | 8.80E-05 | 0.0064716 | 19 |
| GO:1990204 | oxidoreductase complex | 0.0004277 | 0.0065663 | 31 |
| GO:0006909 | phagocytosis | 9.27E-05 | 0.0066706 | 87 |
| GO:1901653 | cellular response to peptide | 9.29E-05 | 0.0066706 | 90 |
| GO:0030137 | COPI-coated vesicle | 0.0004647 | 0.0069891 | 12 |
| GO:0032984 | protein-containing complex disassembly | 0.0001008 | 0.0070281 | 77 |
| GO:0015980 | energy derivation by oxidation of organic compounds | 0.000101 | 0.0070281 | 67 |
| GO:0071219 | cellular response to molecule of bacterial origin | 0.0001012 | 0.0070281 | 56 |
| GO:0005770 | late endosome | 0.0004769 | 0.0070293 | 64 |
| GO:0090383 | phagosome acidification | 0.0001056 | 0.0072525 | 13 |
| GO:0032945 | negative regulation of mononuclear cell proliferation | 0.0001096 | 0.0074 | 26 |
| GO:0048608 | reproductive structure development | 0.0001118 | 0.0074 | 98 |
| GO:0022409 | positive regulation of cell-cell adhesion | 0.0001132 | 0.0074 | 67 |
| GO:0051783 | regulation of nuclear division | 0.0001138 | 0.0074 | 49 |
| GO:0010506 | regulation of autophagy | 0.000114 | 0.0074 | 80 |
| GO:0051250 | negative regulation of lymphocyte activation | 0.0001159 | 0.0074 | 42 |
| GO:0071222 | cellular response to lipopolysaccharide | 0.0001159 | 0.0074 | 53 |
| GO:0000922 | spindle pole | 0.0005182 | 0.007489 | 42 |
| GO:1902107 | positive regulation of leukocyte differentiation | 0.0001191 | 0.007531 | 43 |
| GO:0033047 | regulation of mitotic sister chromatid segregation | 0.0001276 | 0.0079851 | 24 |
| GO:0034399 | nuclear periphery | 0.0005684 | 0.0080561 | 35 |
| GO:1903708 | positive regulation of hemopoiesis | 0.0001324 | 0.0081491 | 52 |
| GO:0051348 | negative regulation of transferase activity | 0.0001328 | 0.0081491 | 70 |
| GO:0090575 | RNA polymerase II transcription regulator complex | 0.000594 | 0.0082598 | 41 |
| GO:0030139 | endocytic vesicle | 0.0006361 | 0.0086819 | 70 |
| GO:0042326 | negative regulation of phosphorylation | 0.0001442 | 0.0087627 | 105 |
| GO:0046631 | alpha-beta T cell activation | 0.0001496 | 0.00895 | 40 |
| GO:0150076 | neuroinflammatory response | 0.0001501 | 0.00895 | 25 |
| GO:0000082 | G1/S transition of mitotic cell cycle | 0.0001548 | 0.0089557 | 68 |
| GO:0002456 | T cell mediated immunity | 0.000156 | 0.0089557 | 31 |
| GO:0070664 | negative regulation of leukocyte proliferation | 0.0001572 | 0.0089557 | 27 |
| GO:0071902 | positive regulation of protein serine/threonine kinase activity | 0.0001583 | 0.0089557 | 79 |
| GO:0061458 | reproductive system development | 0.0001583 | 0.0089557 | 98 |
| GO:0002709 | regulation of T cell mediated immunity | 0.0001625 | 0.0089557 | 24 |
| GO:0051047 | positive regulation of secretion | 0.000163 | 0.0089557 | 78 |
| GO:0090382 | phagosome maturation | 0.0001635 | 0.0089557 | 18 |
| GO:0030183 | B cell differentiation | 0.0001639 | 0.0089557 | 38 |
| GO:0045577 | regulation of B cell differentiation | 0.0001644 | 0.0089557 | 13 |
| GO:0005942 | phosphatidylinositol 3-kinase complex | 0.0006825 | 0.0089908 | 12 |
| GO:0030055 | cell-substrate junction | 0.0006832 | 0.0089908 | 90 |
| GO:0071241 | cellular response to inorganic substance | 0.0001694 | 0.0091529 | 55 |
| GO:0000819 | sister chromatid segregation | 0.0001727 | 0.0091734 | 50 |
| GO:0071375 | cellular response to peptide hormone stimulus | 0.0001727 | 0.0091734 | 76 |
| GO:0061640 | cytoskeleton-dependent cytokinesis | 0.0001742 | 0.0091784 | 30 |
| GO:0030127 | COPII vesicle coat | 0.0007206 | 0.0092117 | 8 |
| GO:0043596 | nuclear replication fork | 0.0007249 | 0.0092117 | 15 |
| GO:0006271 | DNA strand elongation involved in DNA replication | 0.0001839 | 0.0096052 | 10 |
| GO:0017157 | regulation of exocytosis | 0.0001919 | 0.0098477 | 55 |
| GO:0000045 | autophagosome assembly | 0.0001943 | 0.0098477 | 29 |
| GO:0006890 | retrograde vesicle-mediated transport, Golgi to endoplasmic reticulum | 0.0001947 | 0.0098477 | 27 |
| GO:0048013 | ephrin receptor signaling pathway | 0.0001947 | 0.0098477 | 27 |
| GO:0090150 | establishment of protein localization to membrane | 0.0001984 | 0.009878 | 78 |
| GO:0010965 | regulation of mitotic sister chromatid separation | 0.0001985 | 0.009878 | 21 |
| GO:0001503 | ossification | 0.0002041 | 0.0100803 | 91 |
| GO:0022616 | DNA strand elongation | 0.000209 | 0.01024 | 12 |
| GO:0042116 | macrophage activation | 0.0002117 | 0.0102933 | 30 |
| GO:0002699 | positive regulation of immune effector process | 0.000217 | 0.0104721 | 55 |
| GO:0045931 | positive regulation of mitotic cell cycle | 0.0002201 | 0.0105245 | 44 |
| GO:0042059 | negative regulation of epidermal growth factor receptor signaling pathway | 0.0002214 | 0.0105245 | 18 |
| GO:1904951 | positive regulation of establishment of protein localization | 0.0002248 | 0.0106056 | 83 |
| GO:0050000 | chromosome localization | 0.0002362 | 0.0108964 | 25 |
| GO:0050672 | negative regulation of lymphocyte proliferation | 0.0002362 | 0.0108964 | 25 |
| GO:0051303 | establishment of chromosome localization | 0.0002362 | 0.0108964 | 25 |
| GO:1903828 | negative regulation of cellular protein localization | 0.0002576 | 0.0118011 | 33 |
| GO:0000118 | histone deacetylase complex | 0.0009485 | 0.0118485 | 23 |
| GO:0006297 | nucleotide-excision repair, DNA gap filling | 0.0002608 | 0.0118613 | 11 |
| GO:0038127 | ERBB signaling pathway | 0.0002722 | 0.0122007 | 39 |
| GO:0016050 | vesicle organization | 0.0002734 | 0.0122007 | 77 |
| GO:0006891 | intra-Golgi vesicle-mediated transport | 0.0002776 | 0.0122007 | 14 |
| GO:0007034 | vacuolar transport | 0.0002779 | 0.0122007 | 40 |
| GO:0045580 | regulation of T cell differentiation | 0.0002779 | 0.0122007 | 40 |
| GO:1903706 | regulation of hemopoiesis | 0.0002853 | 0.0124351 | 106 |
| GO:0000775 | chromosome, centromeric region | 0.0010276 | 0.0126222 | 47 |
| hsa04611 | Platelet activation | 0.0003163 | 0.0130475 | 38 |
| GO:0006706 | steroid catabolic process | 0.0003235 | 0.0140054 | 12 |
| hsa00520 | Amino sugar and nucleotide sugar metabolism | 0.0004252 | 0.0141277 | 19 |
| hsa05220 | Chronic myeloid leukemia | 0.0004281 | 0.0141277 | 26 |
| GO:0031349 | positive regulation of defense response | 0.0003294 | 0.0141662 | 85 |
| GO:0033673 | negative regulation of kinase activity | 0.0003324 | 0.0141955 | 63 |
| GO:0016241 | regulation of macroautophagy | 0.0003362 | 0.0142604 | 45 |
| GO:1905037 | autophagosome organization | 0.0003471 | 0.0146262 | 29 |
| GO:0042058 | regulation of epidermal growth factor receptor signaling pathway | 0.0003596 | 0.015054 | 27 |
| GO:1901215 | negative regulation of neuron death | 0.0003657 | 0.0151156 | 52 |
| GO:0006892 | post-Golgi vesicle-mediated transport | 0.0003706 | 0.0151156 | 30 |
| GO:0051222 | positive regulation of protein transport | 0.0003717 | 0.0151156 | 79 |
| GO:0033157 | regulation of intracellular protein transport | 0.0003789 | 0.0151156 | 62 |
| GO:0006707 | cholesterol catabolic process | 0.0003879 | 0.0151156 | 7 |
| GO:0016078 | tRNA catabolic process | 0.0003879 | 0.0151156 | 7 |
| GO:0016127 | sterol catabolic process | 0.0003879 | 0.0151156 | 7 |
| GO:0007548 | sex differentiation | 0.0003897 | 0.0151156 | 65 |
| GO:0006903 | vesicle targeting | 0.0003898 | 0.0151156 | 28 |
| GO:0051668 | localization within membrane | 0.0003898 | 0.0151156 | 28 |
| GO:0043434 | response to peptide hormone | 0.0003918 | 0.0151156 | 96 |
| GO:0060249 | anatomical structure homeostasis | 0.0003919 | 0.0151156 | 100 |
| GO:0006979 | response to oxidative stress | 0.0003922 | 0.0151156 | 98 |
| GO:0001914 | regulation of T cell mediated cytotoxicity | 0.0003973 | 0.0152202 | 14 |
| GO:0030120 | vesicle coat | 0.0013057 | 0.0157689 | 18 |
| GO:0001891 | phagocytic cup | 0.0013266 | 0.0157689 | 11 |
| GO:0051306 | mitotic sister chromatid separation | 0.0004202 | 0.0160009 | 21 |
| GO:0090068 | positive regulation of cell cycle process | 0.0004251 | 0.0160879 | 69 |
| GO:0046660 | female sex differentiation | 0.0004291 | 0.0160879 | 33 |
| GO:0071248 | cellular response to metal ion | 0.0004314 | 0.0160879 | 48 |
| GO:0051897 | positive regulation of protein kinase B signaling | 0.0004387 | 0.0160879 | 45 |
| GO:0048285 | organelle fission | 0.0004397 | 0.0160879 | 101 |
| GO:1903532 | positive regulation of secretion by cell | 0.0004405 | 0.0160879 | 71 |
| GO:0045137 | development of primary sexual characteristics | 0.000441 | 0.0160879 | 55 |
| GO:0006283 | transcription-coupled nucleotide-excision repair | 0.0004429 | 0.0160879 | 23 |
| GO:0000209 | protein polyubiquitination | 0.0004512 | 0.0161026 | 76 |
| GO:1990778 | protein localization to cell periphery | 0.0004512 | 0.0161026 | 76 |
| GO:0044784 | metaphase/anaphase transition of cell cycle | 0.0004533 | 0.0161026 | 20 |
| GO:0051098 | regulation of binding | 0.0004535 | 0.0161026 | 81 |
| GO:0043523 | regulation of neuron apoptotic process | 0.0004643 | 0.0163789 | 52 |
| GO:0006289 | nucleotide-excision repair | 0.0004664 | 0.0163789 | 31 |
| GO:0034470 | ncRNA processing | 0.0004703 | 0.0164245 | 87 |
| GO:0051225 | spindle assembly | 0.0004871 | 0.0169025 | 32 |
| GO:0051453 | regulation of intracellular pH | 0.0004893 | 0.0169025 | 26 |
| GO:0051495 | positive regulation of cytoskeleton organization | 0.000494 | 0.0169649 | 55 |
| GO:0000280 | nuclear division | 0.0004965 | 0.0169649 | 92 |
| hsa05110 | Vibrio cholerae infection | 0.000573 | 0.0171888 | 19 |
| GO:0017119 | Golgi transport complex | 0.0014696 | 0.0171919 | 7 |
| GO:0005635 | nuclear envelope | 0.0015279 | 0.0175945 | 97 |
| GO:0033179 | proton-transporting V-type ATPase, V0 domain | 0.001578 | 0.0178921 | 6 |
| GO:0032588 | trans-Golgi network membrane | 0.0016092 | 0.0179696 | 26 |
| GO:0034599 | cellular response to oxidative stress | 0.0005506 | 0.0186007 | 70 |
| GO:1905521 | regulation of macrophage migration | 0.0005573 | 0.0186007 | 16 |
| GO:0002755 | MyD88-dependent toll-like receptor signaling pathway | 0.0005584 | 0.0186007 | 14 |
| GO:0009595 | detection of biotic stimulus | 0.0005584 | 0.0186007 | 14 |
| GO:0043001 | Golgi to plasma membrane protein transport | 0.0005648 | 0.0186007 | 15 |
| GO:0046634 | regulation of alpha-beta T cell activation | 0.000565 | 0.0186007 | 28 |
| GO:1901184 | regulation of ERBB signaling pathway | 0.000565 | 0.0186007 | 28 |
| GO:0030509 | BMP signaling pathway | 0.0005718 | 0.0187277 | 40 |
| GO:0032755 | positive regulation of interleukin-6 production | 0.0005939 | 0.0193501 | 26 |
| hsa05205 | Proteoglycans in cancer | 0.0007586 | 0.0208603 | 55 |
| GO:0046822 | regulation of nucleocytoplasmic transport | 0.000653 | 0.0210775 | 31 |
| GO:0051170 | import into nucleus | 0.0006537 | 0.0210775 | 43 |
| GO:0000723 | telomere maintenance | 0.0006569 | 0.0210775 | 41 |
| GO:0048010 | vascular endothelial growth factor receptor signaling pathway | 0.0006761 | 0.0215837 | 28 |
| GO:0050690 | regulation of defense response to virus by virus | 0.0007147 | 0.0227002 | 12 |
| GO:2001252 | positive regulation of chromosome organization | 0.0007309 | 0.0230916 | 45 |
| GO:0006605 | protein targeting | 0.0007343 | 0.0230916 | 94 |
| GO:0000910 | cytokinesis | 0.0007435 | 0.0232654 | 43 |
| GO:0008022 | protein C-terminus binding | 5.67E-05 | 0.0234101 | 52 |
| GO:0005518 | collagen binding | 5.81E-05 | 0.0234101 | 25 |
| GO:0044769 | ATPase activity, coupled to transmembrane movement of ions, rotational mechanism | 8.10E-05 | 0.0234101 | 11 |
| GO:0046961 | proton-transporting ATPase activity, rotational mechanism | 8.10E-05 | 0.0234101 | 11 |
| GO:0035325 | Toll-like receptor binding | 0.0001274 | 0.0234893 | 8 |
| GO:0008094 | DNA-dependent ATPase activity | 0.0001496 | 0.0234893 | 33 |
| GO:0017116 | single-stranded DNA helicase activity | 0.0001547 | 0.0234893 | 10 |
| GO:0019003 | GDP binding | 0.0001626 | 0.0234893 | 25 |
| GO:0016887 | ATPase activity | 0.0001861 | 0.0235658 | 97 |
| GO:0004386 | helicase activity | 0.0002055 | 0.0235658 | 44 |
| GO:0071723 | lipopeptide binding | 0.0002242 | 0.0235658 | 7 |
| GO:0030641 | regulation of cellular pH | 0.0007641 | 0.023792 | 27 |
| GO:0050868 | negative regulation of T cell activation | 0.0007917 | 0.02428 | 32 |
| GO:0007091 | metaphase/anaphase transition of mitotic cell cycle | 0.0007949 | 0.02428 | 19 |
| GO:1902099 | regulation of metaphase/anaphase transition of cell cycle | 0.0007949 | 0.02428 | 19 |
| GO:0045930 | negative regulation of mitotic cell cycle | 0.0008 | 0.02428 | 75 |
| GO:0043624 | cellular protein complex disassembly | 0.0008043 | 0.02428 | 53 |
| GO:0031987 | locomotion involved in locomotory behavior | 0.0008066 | 0.02428 | 7 |
| GO:0032490 | detection of molecule of bacterial origin | 0.0008066 | 0.02428 | 7 |
| GO:0031253 | cell projection membrane | 0.0022259 | 0.0244846 | 73 |
| GO:0006622 | protein targeting to lysosome | 0.0008242 | 0.0246907 | 10 |
| GO:0007040 | lysosome organization | 0.0008325 | 0.0247038 | 21 |
| GO:0080171 | lytic vacuole organization | 0.0008325 | 0.0247038 | 21 |
| GO:0002819 | regulation of adaptive immune response | 0.0008506 | 0.0251249 | 42 |
| GO:0030168 | platelet activation | 0.0008585 | 0.0251423 | 40 |
| GO:1901185 | negative regulation of ERBB signaling pathway | 0.0008592 | 0.0251423 | 18 |
| GO:0006469 | negative regulation of protein kinase activity | 0.0008994 | 0.0261995 | 57 |
| GO:0044843 | cell cycle G1/S phase transition | 0.0009062 | 0.0262763 | 69 |
| GO:0043138 | 3'-5' DNA helicase activity | 0.0002791 | 0.0263406 | 10 |
| GO:0061650 | ubiquitin-like protein conjugating enzyme activity | 0.0002962 | 0.0263406 | 17 |
| GO:0046635 | positive regulation of alpha-beta T cell activation | 0.0009168 | 0.0264545 | 20 |
| GO:0032608 | interferon-beta production | 0.0009207 | 0.0264545 | 17 |
| GO:0001540 | amyloid-beta binding | 0.0003277 | 0.0270553 | 25 |
| GO:0045239 | tricarboxylic acid cycle enzyme complex | 0.0025506 | 0.027502 | 7 |
| GO:1905354 | exoribonuclease complex | 0.0025748 | 0.027502 | 10 |
| GO:0006112 | energy reserve metabolic process | 0.0009732 | 0.0278363 | 25 |
| GO:0032675 | regulation of interleukin-6 production | 0.0009789 | 0.0278732 | 40 |
| GO:1901990 | regulation of mitotic cell cycle phase transition | 0.0009924 | 0.0280808 | 94 |
| GO:0045621 | positive regulation of lymphocyte differentiation | 0.0009951 | 0.0280808 | 29 |
| GO:0062197 | cellular response to chemical stress | 0.0010056 | 0.0282495 | 78 |
| GO:0050766 | positive regulation of phagocytosis | 0.0010315 | 0.0286046 | 21 |
| GO:0032930 | positive regulation of superoxide anion generation | 0.0010462 | 0.0286046 | 9 |
| GO:0050867 | positive regulation of cell activation | 0.0010479 | 0.0286046 | 89 |
| GO:0032733 | positive regulation of interleukin-10 production | 0.0010499 | 0.0286046 | 14 |
| GO:0071276 | cellular response to cadmium ion | 0.0010499 | 0.0286046 | 14 |
| GO:0090224 | regulation of spindle organization | 0.0010499 | 0.0286046 | 14 |
| GO:1905898 | positive regulation of response to endoplasmic reticulum stress | 0.0010499 | 0.0286046 | 14 |
| hsa05323 | Rheumatoid arthritis | 0.0011494 | 0.0291782 | 29 |
| GO:0032635 | interleukin-6 production | 0.0010945 | 0.0296917 | 42 |
| GO:0001933 | negative regulation of protein phosphorylation | 0.0011136 | 0.0300815 | 93 |
| GO:0002221 | pattern recognition receptor signaling pathway | 0.0011192 | 0.0301054 | 48 |
| GO:0042063 | gliogenesis | 0.0011244 | 0.0301158 | 68 |
| GO:0090114 | COPII-coated vesicle budding | 0.0011375 | 0.0303369 | 22 |
| GO:0046686 | response to cadmium ion | 0.0011425 | 0.030344 | 20 |
| GO:0002696 | positive regulation of leukocyte activation | 0.0011837 | 0.0313065 | 86 |
| GO:0003678 | DNA helicase activity | 0.0004088 | 0.0315055 | 25 |
| GO:0000781 | chromosome, telomeric region | 0.0030612 | 0.0321178 | 39 |
| GO:0030133 | transport vesicle | 0.0031101 | 0.0321178 | 84 |
| GO:0016471 | vacuolar proton-transporting V-type ATPase complex | 0.0031813 | 0.0321178 | 8 |
| GO:0030663 | COPI-coated vesicle membrane | 0.0031813 | 0.0321178 | 8 |
| GO:0031341 | regulation of cell killing | 0.0012317 | 0.0324401 | 26 |
| GO:0032653 | regulation of interleukin-10 production | 0.0012605 | 0.0326329 | 19 |
| GO:0032732 | positive regulation of interleukin-1 production | 0.0012605 | 0.0326329 | 19 |
| GO:0061951 | establishment of protein localization to plasma membrane | 0.0012605 | 0.0326329 | 19 |
| GO:0031570 | DNA integrity checkpoint | 0.0012651 | 0.0326329 | 40 |
| GO:0008406 | gonad development | 0.0012672 | 0.0326329 | 52 |
| GO:0050854 | regulation of antigen receptor-mediated signaling pathway | 0.00127 | 0.0326329 | 21 |
| GO:1901216 | positive regulation of neuron death | 0.0012868 | 0.0328839 | 27 |
| GO:0071380 | cellular response to prostaglandin E stimulus | 0.0012902 | 0.0328839 | 8 |
| hsa04218 | Cellular senescence | 0.0015497 | 0.0341143 | 43 |
| hsa04662 | B cell receptor signaling pathway | 0.0015506 | 0.0341143 | 26 |
| GO:0090317 | negative regulation of intracellular protein transport | 0.0013471 | 0.0341965 | 15 |
| hsa05132 | Salmonella infection | 0.0016687 | 0.0344177 | 63 |
| GO:0030071 | regulation of mitotic metaphase/anaphase transition | 0.0013838 | 0.0346298 | 18 |
| GO:0043405 | regulation of MAP kinase activity | 0.0013845 | 0.0346298 | 74 |
| GO:0032481 | positive regulation of type I interferon production | 0.0013861 | 0.0346298 | 22 |
| GO:0036465 | synaptic vesicle recycling | 0.0013861 | 0.0346298 | 22 |
| GO:0048278 | vesicle docking | 0.0014141 | 0.035191 | 20 |
| GO:0006310 | DNA recombination | 0.0014438 | 0.0357897 | 66 |
| hsa04933 | AGE-RAGE signaling pathway in diabetic complications | 0.0018878 | 0.0359854 | 30 |
| hsa04068 | FoxO signaling pathway | 0.0020097 | 0.0359854 | 37 |
| hsa00640 | Propanoate metabolism | 0.0020719 | 0.0359854 | 13 |
| GO:0046546 | development of primary male sexual characteristics | 0.0014735 | 0.0363835 | 36 |
| GO:0044262 | cellular carbohydrate metabolic process | 0.0014967 | 0.0368116 | 65 |
| GO:0045579 | positive regulation of B cell differentiation | 0.0015149 | 0.0371142 | 7 |
| GO:0006986 | response to unfolded protein | 0.0015212 | 0.037125 | 44 |
| GO:0051310 | metaphase plate congression | 0.0015693 | 0.0381529 | 19 |
| GO:0140029 | exocytic process | 0.0015818 | 0.0383092 | 24 |
| GO:0071772 | response to BMP | 0.0015997 | 0.0384265 | 41 |
| GO:0071773 | cellular response to BMP stimulus | 0.0015997 | 0.0384265 | 41 |
| GO:0044774 | mitotic DNA integrity checkpoint | 0.0016049 | 0.0384265 | 29 |
| GO:0002291 | T cell activation via T cell receptor contact with antigen bound to MHC molecule on antigen presenting cell | 0.0016211 | 0.0384427 | 6 |
| GO:0009396 | folic acid-containing compound biosynthetic process | 0.0016211 | 0.0384427 | 6 |
| GO:0018212 | peptidyl-tyrosine modification | 0.0016305 | 0.0384427 | 80 |
| GO:0010038 | response to metal ion | 0.0016344 | 0.0384427 | 78 |
| GO:0032648 | regulation of interferon-beta production | 0.001636 | 0.0384427 | 16 |
| GO:0007252 | I-kappaB phosphorylation | 0.001643 | 0.0384639 | 9 |
| GO:0061631 | ubiquitin conjugating enzyme activity | 0.0005334 | 0.0385413 | 16 |
| GO:0006661 | phosphatidylinositol biosynthetic process | 0.0016584 | 0.0386827 | 31 |
| GO:0008286 | insulin receptor signaling pathway | 0.0016818 | 0.0389629 | 36 |
| GO:0000724 | double-strand break repair via homologous recombination | 0.0016875 | 0.0389629 | 35 |
| GO:0042113 | B cell activation | 0.0016889 | 0.0389629 | 71 |
| GO:0032869 | cellular response to insulin stimulus | 0.0017283 | 0.0392521 | 52 |
| GO:0070534 | protein K63-linked ubiquitination | 0.0017346 | 0.0392521 | 18 |
| GO:1905517 | macrophage migration | 0.0017346 | 0.0392521 | 18 |
| GO:0035966 | response to topologically incorrect protein | 0.0017375 | 0.0392521 | 48 |
| GO:0048207 | vesicle targeting, rough ER to cis-Golgi | 0.0017387 | 0.0392521 | 20 |
| GO:0048208 | COPII vesicle coating | 0.0017387 | 0.0392521 | 20 |
| GO:0001913 | T cell mediated cytotoxicity | 0.0017556 | 0.0394921 | 15 |
| GO:0007059 | chromosome segregation | 0.0017647 | 0.0395556 | 72 |
| GO:0032200 | telomere organization | 0.0017722 | 0.0395729 | 42 |
| GO:0010717 | regulation of epithelial to mesenchymal transition | 0.0017864 | 0.0395729 | 27 |
| GO:0032204 | regulation of telomere maintenance | 0.0017905 | 0.0395729 | 23 |
| GO:1901224 | positive regulation of NIK/NF-kappaB signaling | 0.0017905 | 0.0395729 | 23 |
| hsa05417 | Lipid and atherosclerosis | 0.002465 | 0.0396388 | 55 |
| hsa05163 | Human cytomegalovirus infection | 0.0026298 | 0.0396388 | 57 |
| hsa05142 | Chagas disease | 0.0026426 | 0.0396388 | 30 |
| GO:0051092 | positive regulation of NF-kappaB transcription factor activity | 0.0018569 | 0.0403794 | 39 |
| GO:0044247 | cellular polysaccharide catabolic process | 0.0018597 | 0.0403794 | 10 |
| GO:0090169 | regulation of spindle assembly | 0.0018597 | 0.0403794 | 10 |
| GO:0098581 | detection of external biotic stimulus | 0.0018597 | 0.0403794 | 10 |
| GO:0097352 | autophagosome maturation | 0.0018613 | 0.0403794 | 14 |
| GO:0070507 | regulation of microtubule cytoskeleton organization | 0.0018679 | 0.0403794 | 45 |
| GO:0033627 | cell adhesion mediated by integrin | 0.0018907 | 0.0403794 | 21 |
| GO:0032210 | regulation of telomere maintenance via telomerase | 0.0019079 | 0.0403794 | 17 |
| GO:0046488 | phosphatidylinositol metabolic process | 0.0019099 | 0.0403794 | 44 |
| GO:0051251 | positive regulation of lymphocyte activation | 0.001916 | 0.0403794 | 76 |
| GO:0071706 | tumor necrosis factor superfamily cytokine production | 0.0019168 | 0.0403794 | 31 |
| GO:0007265 | Ras protein signal transduction | 0.0019176 | 0.0403794 | 74 |
| GO:0051101 | regulation of DNA binding | 0.0019288 | 0.0403794 | 32 |
| GO:0030888 | regulation of B cell proliferation | 0.00194 | 0.0403794 | 19 |
| GO:0070527 | platelet aggregation | 0.00194 | 0.0403794 | 19 |
| GO:0033572 | transferrin transport | 0.001942 | 0.0403794 | 13 |
| GO:0042554 | superoxide anion generation | 0.001942 | 0.0403794 | 13 |
| GO:0071634 | regulation of transforming growth factor beta production | 0.001942 | 0.0403794 | 13 |
| GO:0032388 | positive regulation of intracellular transport | 0.0019696 | 0.0406134 | 51 |
| GO:0097529 | myeloid leukocyte migration | 0.0019696 | 0.0406134 | 51 |
| GO:0043161 | proteasome-mediated ubiquitin-dependent protein catabolic process | 0.0019725 | 0.0406134 | 88 |
| GO:0018108 | peptidyl-tyrosine phosphorylation | 0.0019808 | 0.0406518 | 79 |
| GO:0090501 | RNA phosphodiester bond hydrolysis | 0.0020633 | 0.042208 | 40 |
| GO:0045807 | positive regulation of endocytosis | 0.0020933 | 0.0426825 | 27 |
| GO:0007250 | activation of NF-kappaB-inducing kinase activity | 0.0021086 | 0.04272 | 8 |
| GO:0030540 | female genitalia development | 0.0021086 | 0.04272 | 8 |
| GO:0048194 | Golgi vesicle budding | 0.0021411 | 0.0431018 | 23 |
| GO:0071158 | positive regulation of cell cycle arrest | 0.0021411 | 0.0431018 | 23 |
| GO:0005178 | integrin binding | 0.0006348 | 0.0431661 | 39 |
| GO:0006900 | vesicle budding from membrane | 0.0021709 | 0.0435636 | 29 |
| GO:0000725 | recombinational repair | 0.0021942 | 0.0438906 | 35 |
| GO:0022604 | regulation of cell morphogenesis | 0.0022387 | 0.0443911 | 101 |
| GO:0009791 | post-embryonic development | 0.0022403 | 0.0443911 | 24 |
| GO:0034109 | homotypic cell-cell adhesion | 0.0022403 | 0.0443911 | 24 |
| GO:0032365 | intracellular lipid transport | 0.0022625 | 0.0445513 | 15 |
| GO:0072583 | clathrin-dependent endocytosis | 0.0022625 | 0.0445513 | 15 |
| GO:0050663 | cytokine secretion | 0.0022871 | 0.0448962 | 21 |
| GO:0017124 | SH3 domain binding | 0.0007519 | 0.0448962 | 36 |
| GO:0019902 | phosphatase binding | 0.0008017 | 0.0448962 | 49 |
| GO:0140097 | catalytic activity, acting on DNA | 0.0008212 | 0.0448962 | 52 |
| GO:0019787 | ubiquitin-like protein transferase activity | 0.0008478 | 0.0448962 | 100 |
| GO:0003924 | GTPase activity | 0.0008544 | 0.0448962 | 75 |
| GO:0005771 | multivesicular body | 0.0045566 | 0.0453812 | 18 |
| GO:0051648 | vesicle localization | 0.0023304 | 0.0456054 | 52 |
| hsa05152 | Tuberculosis | 0.0032012 | 0.0459301 | 47 |
| GO:0016651 | oxidoreductase activity, acting on NAD(P)H | 0.0009164 | 0.0460576 | 31 |
| GO:0032868 | response to insulin | 0.0023765 | 0.046325 | 62 |
| GO:0032613 | interleukin-10 production | 0.0023818 | 0.046325 | 19 |
| GO:0008360 | regulation of cell shape | 0.0024006 | 0.046547 | 38 |
| GO:0070317 | negative regulation of G0 to G1 transition | 0.0024299 | 0.0469396 | 14 |
| GO:0000077 | DNA damage checkpoint | 0.0024357 | 0.0469396 | 37 |
| GO:0044773 | mitotic DNA damage checkpoint | 0.0024441 | 0.0469579 | 27 |
| GO:0032288 | myelin assembly | 0.0024841 | 0.0474387 | 9 |
| GO:0046641 | positive regulation of alpha-beta T cell proliferation | 0.0024841 | 0.0474387 | 9 |
| GO:0008584 | male gonad development | 0.0024934 | 0.0474716 | 35 |
| GO:0031098 | stress-activated protein kinase signaling cascade | 0.0025158 | 0.0477541 | 65 |
| GO:0032640 | tumor necrosis factor production | 0.0025307 | 0.0478943 | 30 |
| GO:0032688 | negative regulation of interferon-beta production | 0.0026272 | 0.0495715 | 7 |

GO, Gene Ontology; KEGG, Kyoto Encyclopedia of Genes and Genomes; DEGs, differentially expressed genes; FDR, false discovery rate.
