# Supplementary material for: RB1 Is an Immune-Related Prognostic Biomarker for Ovarian Cancer
Source: Front Oncol. 2022 Mar 1;12:830908. doi: 10.3389/fonc.2022.830908 (PMC8920998; doi:10.3389/fonc.2022.830908)
Supplement: Supplementary file 7 [file Table_3.docx]

**Supplementary Table 3. Clinic data of ovarian cancer samples from tissue microarray.**

| **Variable** | **Cases n (%)** | ***P*-value** |
| --- | --- | --- |
| **Total** | 36 (100%) |  |
| **Age** |  |  |
| ≤ 60 | 11 (30.56%) | 0.55 |
| > 60 | 25 (69.44%) |  |
| **TNM stage** |  |  |
| I/II | 13 (36.11%) | 0.23 |
| III/IV | 23 (63.89%) |  |
| **T classification** |  |  |
| T1/T2 | 15 (41.67%) | 0.14 |
| T3 | 21 (58.33%) |  |
| **N classification** |  |  |
| N0 | 23 (63.89%) | 0.65 |
| N1  **M classification** | 13 (36.11%) |  |
| M0  M1  **Histological subtype** | 32 (88.89%)  4 (11.11%) | 0.051 |
| Serous | 27 (75.00%) | 0.0093 |
| Endometrioid | 7 (19.44%) |  |
| Clear-cell carcinoma | 1 (2.78%) |  |
| Endometrioid and serous | 1 (2.78%) |  |
| **Recurrence of state** |  |  |
| Recurrence | 23 (63.89%) | 0.56 |
| No recurrence | 13 (36.11%) |  |
